# Prognostic Value of Neurofilament Light Chain and Glial Fibrillary Acidic Protein in ALD‐Related Myelopathy

**DOI:** 10.1002/acn3.70386

**Published:** 2026-04-08

**Authors:** Eda G. Kabak, Marije M. C. Voermans, Hans Heijst, Charlotte E. Teunissen, Marc Engelen

**Affiliations:** ^1^ Department of Neurology and Pediatric Neurology Emma Children's Hospital, Amsterdam Leukodystrophy Center, Amsterdam University Medical Center Amsterdam AZ the Netherlands; ^2^ Neurochemistry Laboratory, Department of Clinical Chemistry Amsterdam Neuroscience, Vrije Universiteit Amsterdam Amsterdam HV the Netherlands; ^3^ Amsterdam Neuroscience, Neurodegeneration Amsterdam HV the Netherlands

**Keywords:** GFAP, myelopathy, NfL, X‐ALD

## Abstract

**Background:**

X‐linked adrenoleukodystrophy (X‐ALD) is a neurometabolic disorder caused by pathogenic variants in *ABCD1*, leading to slowly progressive spinal cord disease in nearly all affected men. Sensitive biomarkers to quantify disease severity and predict progression are needed for clinical care and trial design. Plasma neurofilament light chain (NfL) and glial fibrillary acidic protein (GFAP) are promising biomarkers reflecting axonal and astroglial injury. This study evaluated their prognostic value for spinal cord disease progression in X‐ALD.

**Methods:**

In a prospective, seven‐year longitudinal study, 66 adult male X‐ALD patients without cerebral involvement were followed. Plasma NfL and GFAP were measured using single‐molecule array (Simoa) technology. Patients were stratified by baseline biomarker levels using cohort‐based fourth‐quartile cut‐offs (NfL < 15.7 vs. ≥ 15.7 pg/mL; GFAP < 78.7 vs. ≥ 78.7 pg/mL). Age‐adjusted NfL residuals were calculated from baseline log‐transformed NfL regressed on age. Longitudinal trajectories of the EDSS, SSPROM, and 6‐MWT were analyzed using linear mixed‐effect models.

**Results:**

High baseline NfL was associated with faster EDSS progression (*p* < 0.001) and steeper SSPROM decline, with differences emerging within the first year. Overall plasma NfL levels remained stable over time (*p* = 0.111). Age‐adjusted NfL residuals showed significant slope differences between the mean and high (+1 SD) and very high (+2 SD) groups. GFAP stratification showed limited prognostic value, with only a significant decline in both groups for SSPROM.

**Interpretation:**

Baseline plasma NfL is a robust prognostic biomarker for spinal cord disease progression in adult X‐ALD and supports its use for patient stratification in clinical trials, whereas GFAP shows limited standalone utility.

## 1 Introduction

X‐linked adrenoleukodystrophy (X‐ALD) is a neurometabolic disorder caused by pathogenic variants in the ABCD‐1 gene, leading to the accumulation of very long‐chain fatty acids (VLCFA; ≥C26:0). Patients develop progressive neurodegeneration of the central and peripheral nervous system, and adrenal insufficiency [1, 2]. A leukodystrophy affects approximately 60% of male patients. All males and most female patients develop slowly progressive spinal cord disease that predominantly affects the dorsal columns and the corticospinal tracts [3, 4, 5]. Changes in the severity of spinal cord disease are often difficult to quantify with current clinimetric tools [6, 7]. Sensitive biomarkers to monitor disease activity and predict progression are needed.

Neurofilament light chain (NfL), a cytoskeletal protein released into the cerebrospinal fluid (CSF) and blood following axonal damage [8], is a potential prognostic marker for neurodegeneration in X‐ALD. Similarly, glial fibrillary acidic protein (GFAP), an intermediate filament protein specific to astrocytes, serving as a marker of astroglial injury or activity [9], has gained interest in several neurodegenerative and neuroinflammatory diseases [10, 11, 12]. Ultra‐sensitive immunoassays such as single‐molecule array (Simoa) technology enable accurate and routine quantification of both NfL and GFAP in plasma or serum and are increasingly implemented in clinical settings [13, 14, 15, 16].

In ALD, it has been shown that NfL concentrations in serum and CSF are elevated in adult male patients with X‐ALD, especially in those with advanced spinal cord disease and leukodystrophy. Van Ballegoij et al. [17] showed that there is a correlation between NfL concentration and the severity of spinal cord disease. Longitudinal assessment in the pediatric population with CALD suggests that plasma NfL may also serve as a sensitive tool to monitor lesion development [18]. GFAP has less robust correlation with the severity of spinal cord disease in ALD [17].

The slow disease progression in ALD is a challenge for clinical trials, where traditional outcome measures such as EDSS or 6‐min walk test (6MWT) often require extended follow‐up periods to detect meaningful change, delaying the evaluation of novel therapies [1, 6, 7]. The highly variable rate of disease progression between patients means large numbers of patients are needed. Prognostic biomarkers allow stratification of patients into groups with an expected similar disease course. In this study, we investigate the potential of NfL and GFAP as prognostic biomarkers for ALD‐related spinal cord disease.

## 2 Methods

### 2.1 Study Design and Participants

A prospective, longitudinal study. The Dutch X‐ALD cohort [6, 7, 17, 19] is ongoing. The current study uses data from 2015 to 2024. Patients underwent regular clinical assessment, imaging, and laboratory evaluations. Blood samples were collected at each visit and stored in a biobank for further analyses.

For this study, only men (≥ 18 years) were selected. Patients with leukodystrophy (gadolinium‐enhancing or non‐enhancing), a history of hematopoietic stem cell transplantation (HSCT), gene therapy, or compassionate use of leriglitazone were excluded. Additionally, timepoints with fewer than 10 patients were excluded from the analyses to maintain statistical power, resulting in a maximum of eight timepoints, spanning seven years of follow‐up.

All patients provided informed consent for participation in the study and storage of biomaterials in the biobank prior to inclusion. The protocols of the ongoing study were approved by the local institutional review board of Amsterdam UMC (METC 2018–310).

### 2.2 Assessment of Myelopathy

Patients underwent a standardized neurologic examination. If both symptomatology and signs of myelopathy were present, patients were scored as having a myelopathy. Clinimetric scales were used in the clinical assessments (Expanded Disability Status Scale (EDSS), ranging from 0 (no disability) to 10 (death), and Severity Scoring system for Myelopathy (SSPROM), ranging from 0 to 100 with 100 indicating absence of symptoms). The Opal system (APDM, Portland, OR) was used to assess sway and gait. For this particular study, only 6‐min walking test outcomes of the quantitative gait assessment were used.

### 2.3 Sample Processing

Blood samples were collected from participants in the extended “Dutch X‐ALD cohort” in 4.5‐mL EDTA tubes and processed within 2 h at the biobank of Amsterdam UMC, following protocols similar to those described by van Ballegoij et al. [17]. Briefly, samples were centrifuged at 2000 g for 10 min, and plasma was aliquoted in 0.5 mL volumes and stored at −80°C until analysis.

For the purpose of this study, plasma levels of NfL and GFAP were retrospectively assessed from biobank samples collected prior to 2023. From 2023 onwards, NfL levels were measured directly at the time of sample collection, while GFAP continued to be analyzed retrospectively from biobank specimens.

Measurements of NfL and GFAP in plasma and CSF were conducted at the Neurochemistry Laboratory of Amsterdam UMC, location VUmc, using single‐molecule array (Simoa) technology (Quanterix Corp., MA, USA), as previously detailed by van Ballegoij et al. [17]. Analyses employed the NF‐light Kit (Quanterix) for NfL and the GFAP Discovery Kit (Quanterix), performed on the SiMoA HD‐1 and HD‐X platform according to the manufacturer's protocols (www.quanterix.com/products‐technology/assays). All measurements were conducted by certified technicians blinded to clinical data, with average intra‐assay coefficients of variation of 4.8% for NfL and 3.3% for GFAP, aligning with the quality standards reported by van Ballegoij et al. [17].

### 2.4 Statistical Analyses

#### 2.4.1 Longitudinal NfL and GFAP Assessment

Plasma NfL and GFAP concentrations were analyzed longitudinally to assess the trajectory of biomarker levels over time. Only visits with at least ten patients were included: Baseline up to follow‐up year seven. Linear mixed effect models (LMMs) were used via the “lmer” function. The model included “Visit” as a continuous fixed effect and random intercepts and slopes for “Visit” grouped by subject, accounting for individual variability in GFAP over time. LMMs were fitted using maximum likelihood estimation. Estimated marginal means (EMMs) for each “Visit” level were computed, and pairwise comparisons between visits were performed with FDR adjustment to control for multiple testing. Confidence intervals for predicted GFAP and NfL values were generated, and longitudinal model predictions with 95% confidence intervals were visualized.

#### 2.4.2 Baseline Stratification Based on NfL and GFAP

The median and interquartile ranges of serum NfL of the selected cohort were calculated, and the cut‐off was based on the value of the interquartile ranges. Values in the 4th quartile (Q4) were considered high levels, and levels within the first three quartiles were considered low. Baseline plasma NfL levels were stratified into groups with a threshold of 15.7 pg/mL.

Similarly, baseline GFAP levels were dichotomized based on the cohort median and IQR, with a threshold of 78.7 pg/mL, which was the upper limit of the 3rd quartile.

#### 2.4.3 Baseline Assessment

Baseline statistics were summarized using descriptive statistics. The distribution of continuous variables was assessed for normality using the Kolmogorov–Smirnov test. For normally distributed data, group comparisons were conducted using independent samples *t*‐tests and reported as means ± standard deviations (SDs). Non‐normally distributed data were analyzed using the Wilcoxon rank‐sum test and reported as medians with inter‐quartile ranges (IQR). To control for multiple comparisons, *p*‐values were adjusted using false discovery rate (FDR) correction.

#### 2.4.4 Longitudinal Analyses

Longitudinal trajectories of EDSS, SSPROM, and 6‐MWT were analyzed using LMMs via the “lmer” function. Models included fixed effects for time (modeled continuously in years), biomarker group (NfL or GFAP), and age as a covariate. Random intercepts and slopes for time were included, nested within participants, to account for individual variability. A time × subgroup interaction was added to assess the differences in trajectories of the clinical scales. LMMs were fitted using maximum likelihood estimation. Estimated marginal means (EMMs) were computed at annual intervals from baseline (year 0) to year 7 with corresponding 95% confidence intervals (95% CIs). Pairwise contrasts comparing each follow‐up time point to baseline were performed with FDR *p*‐values to account for multiple testing. Results are presented as estimated means, standard errors, and corrected *p*‐values.

#### 2.4.5 Sensitivity Analyses: Age‐Adjusted NfL

A sensitivity analysis was performed to address potential confounding by age and to isolate disease‐associated elevations in plasma NfL from those attributable to normal aging. Using baseline data, log‐transformed plasma NfL levels were modeled as a function of age within the full study cohort using linear regression. Standardized residuals were subsequently calculated by subtracting the age‐predicted log(NfL) value from the observed log(NfL) value and dividing this difference by the residual standard deviation of the model. This standardized residual reflects higher‐ or lower‐than‐expected‐for‐age NfL within the X‐ALD cohort and is not a normative z‐score derived from an external healthy reference population.

For interpretability and visualization purposes, the age‐adjusted NfL residual was expressed in standard deviation (SD) units and categorized into four groups (−1 SD, 0 SD, +1 SD, +2 SD).

The age‐adjusted NfL residual was analyzed both as a continuous predictor and as grouped SD categories in longitudinal mixed‐effects models assessing EDSS, SSPROM, and 6‐min walking test trajectories. This sensitivity analysis was performed to evaluate whether associations between NfL and clinical progression persisted independently of chronological age.

Statistical significance was set at α = 0.05 (two‐sided). Effect sizes are reported as generalized eta squared (η^2^G), interpreted as small (0.001–0.06), medium (0.06–0.14), and large (> 0.14) effects. Data were analyzed using R version 4.4.0 (R Core Team, 2025).

## 3 Results

### 3.1 Rationale for Baseline Biomarker Stratification

Baseline plasma NfL concentrations in X‐ALD show substantial interindividual variability and are known to increase with age, even in neurologically asymptomatic individuals. Prior studies in healthy adults established different cut‐off values for age ranges [20, 21]. Given the age‐heterogeneous nature of the cohort and the absence of disease‐specific normative reference values, baseline stratification was therefore performed using cohort‐based distributional thresholds, and patients in the highest quartile (Q4) were classified as having high levels of biomarkers.

### 3.2 Baseline Characteristics NfL

At baseline, 66 participants were included, with 53 (80%) in the NfL < 15.7 pg/mL group and 13 (20%) in the NfL ≥ 15.7 pg/mL group. Mean age was significantly higher in the ≥ 15.7 pg/mL group (*p* = 0.003). EDSS scores were significantly higher in the ≥ 15.7 pg/mL group (*p* < 0.001), and SSPROM scores were significantly lower.

(*p* = 0.005). Mean 6‐MWT levels were also significantly lower in the ≥ 15.7 pg/mL group (*p* < 0.001) (Table 1).

**TABLE 1:** Baseline stratified by NfL and GFAP subgroup.

Characteristic	NfL < 15.7 pg/mL(*n* = 53)	NfL ≥ 15.7 pg/mL(*n* = 13)	*p*‐value*	GFAP < 78.7 pg/mL(*n* = 40)	GFAP ≥ 78.7 pg/mL(*n* = 12)	*p*‐value*
Age, years	36.4 (20.3)	58.4 (17.2)	0.003	35.6 (17.4)	56.2 (30.4)	0.014
EDSS	2.94 (1.8)	4.64 (1.1)	< 0.001	3.3 (1.9)	3.4 (1.6)	0.745
SSPROM	88.7 (11.1)	80.2 (5.2)	0.005	87.2 (10.9)	87.5 (11.0)	0870
6‐min walk test, m	529 ± 132	333 ± 84.8	< 0.001	515.2 ± 137.7	463.4 ± 174.5	0.112

*Note:* Data is presented as either mean (SD) or median (IQR).

Abbreviations: EDSS (Expanded Disability Status Scale), SSPROM (Severity Scoring system for Myelopathy), 6‐MWT: 6‐min walk test.

* FDR‐adjusted significance.

### 3.3 Baseline Characteristics GFAP

Of 52 participants with GFAP levels available, 40 patients were in the < 78.7 pg/mL group, and 12 patients were in the ≥ 78.7 pg/mL group. Mean age was significantly higher in the ≥ 78.7 pg/mL group compared to the < 78.7 pg/mL group (56.2 years [SD 30.4] vs. 35.6 years [SD 17.4], *p* = 0.014). No statistically significant differences were observed in mean EDSS scores, SSPROM, or 6‐MWT scores between the groups (Table 1).

### 3.4 NfL Change Over Time

LMMs showed no statistically significant effect of time on NfL plasma levels across visits (*p* = 0.111) (Supplementary Figure 1).

Estimated marginal means ranged from 13.68 pg/mL (Baseline, SE = 1.40) to 19.4 (V7, SE = 1.97). Pairwise comparisons, adjusted for multiple comparisons with FDR correction, showed no statistically significant differences between baseline visit and follow‐up visits except for year 6 vs. baseline visit with a mean change of 5.93 (SE = 1.575, *p* = 0.003) and year 7 vs. baseline with a mean change of 6.54 (SE = 1.80, *p* = 0.002). Detailed results are shown in Supplementary Table 1.

### 3.5 Longitudinal Predictive Value of NfL

**FIGURE 1:**
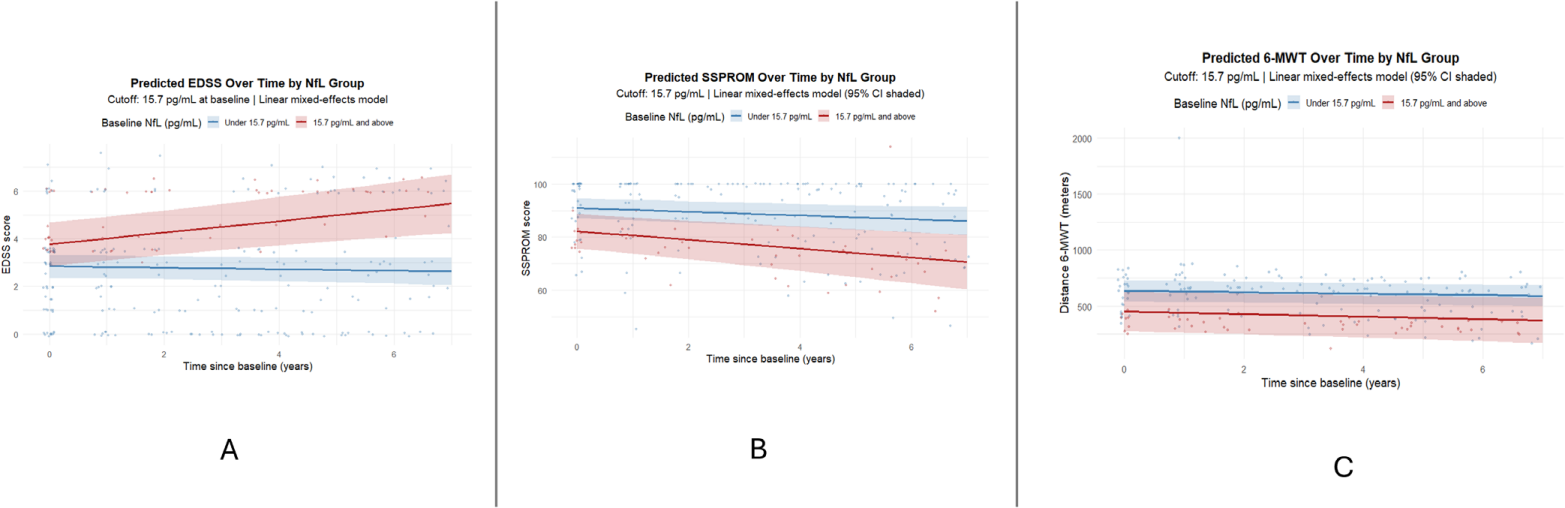
(A−C). Longitudinal Trajectories of clinical outcomes stratified by baseline plasma NfL (15.7 pg/mL cut‐off). Predicted longitudinal trajectories of clinical outcome measures stratified by baseline plasma neurofilament light (NfL) concentration using a cutoff of 15.7 pg/mL. Participants were classified as having low NfL (< 15.7 pg/mL) (*n* = 53) or high NfL (≥ 15.7 pg/mL) (*n* = 13) at baseline. Predictions were obtained from linear mixed‐effects models including time as a continuous variable and random intercepts and slopes for participants, adjusted for age. Solid lines represent model‐estimated mean trajectories, shaded bands indicate 95% confidence intervals, and individual dots represent observed measurements at each visit. Abbreviations: (A) Expanded Disability Status Scale (EDSS), (B) Severity Scoring System for Myelopathy (SSPROM), (C) 6‐Minute Walking Test (6‐MWT). Abbreviations: NfL: Neurofilament Light Chain; pg/mL: Picograms per milliliters; EDSS: Expanded Disability Status Scale; SSPROM: Severity Scoring System for Myelopathy; 6‐MWT: 6‐min walk test.

The analysis of clinical outcome measures over time stratified by NfL subgroups < 15.7 pg/mL and ≥ 15.7 pg/mL showed significant differences in disease progression (Figure 1).

In the NfL ≥ 15.7 pg/mL group, EDSS scores increased significantly over time. This significant progression was evident at the first year of follow‐up (V1–V0, *p* = 0.002), with an estimated mean change of 0.24 (SE 0.06, 95% CI: 0.07–0.44, effect size 0.40) and continued up to V7 (+1.70, SE 0.45, *p* < 0.001, 95% CI: 0.44–2.96, effect size 0.45) (Supplementary Table 2A). In contrast, the NfL < 15.7 pg/mL subgroup showed minimal, non‐significant change over the same period (*p* = 0.322) (Figure 1A). Linear mixed effect models indicated a significant difference in the disability progression slope between the NfL subgroups (β = −0.27, *p* < 0.001) (Table 2).

**TABLE 2:** Comparison of Annual Rates of Change Across Outcomes NfL < 15.7 pg/mL vs. ≥ 15.7 pg/mL.

Outcome	Between‐Group Slope Difference Δ [95% CI]	*p*‐value*
EDSS	−0.270 [−0.419 to −0.151]	< 0.001
SSPROM	0.95 [−1.93 to 0.01]	0.060
6MWT	5.07 [−16.5 to 5.3]	0.317

*Note:* Δ (Between‐Group Slope Difference) is defined as High‐Low (compared to Time × NfL_groupUnder15.7 interaction term).

Abbreviations: EDSS (Expanded Disability Status Scale), SSPROM (Severity Scoring system for Myelopathy), 6‐MWT: 6‐min walk test.

* *p*‐value is for the Time × NfL group interaction.

For SSPROM scores, both NfL groups showed significant declines over time within early follow‐up periods (Figure 1B). The ≥ 15.7 pg/mL group experienced a greater decline, with a change of −1.68 at V1−V0 (SE 0.46, *p* = 0.001, 95% CI: −2.99 to −0.37, effect size 0.43), progressing to −11.62 by V7−V0. The < 15.7 pg/mL group showed a smaller, though still significant, decline of −0.72 at V1−V0 (SE 0.25, *p* = 0.008, 95% CI: −1.41 to 0.007, effect size 0.34), reaching −45.03 by V7−V0 (Supplementary Table 2B). The interaction between time and NfL subgroup was not statistically significant (β = +0.95, *p* = 0.060), indicating that the rate of decline in patient‐reported outcomes did not differ significantly between the groups (Table 2).

For the 6‐MWT, the NfL ≥ 15.7 pg/mL group showed a significant decline over time, whereas this was not shown in the NfL < 15.7 pg/mL group (≥ 15.7 pg/mL: V7−V0 *p* = 0.038; < 15.7 pg/mL: V7−V0 *p* = 0.095) (Figure 1C). The ≥ 15.7 pg/mL group exhibited a decrease of −11.58 m at V1–V0 (SE 5.29, 95% CI: −26.49 to 3.33, effect size 0.18), while the < 15.7 pg/mL group had a decrease of −6.51 m (SE 3.21, 95% CI: −165.45 to 2.42, effect size 0.24) (Supplementary Table 2C). However, the interaction between Time and NfL subgroup was not significant (β = +5.07, *p* = 0.317), indicating that the rate of decline in walking capacity did not differ between groups (Table 2).

### 3.6 Progression Slopes by Age‐Adjusted NfL Group

Higher age‐adjusted NfL residuals were associated with progressively steeper EDSS and SSPROM slopes. Participants in the very high residual group (+2 SD) showed significant EDSS worsening (slope + 0.137 per visit, *p* = 0.002) and SSPROM decline (−1.276 per visit, *p* < 0.001), while no significant progression was observed in the low residual group (Table 3A–C).

**TABLE 3:** Progression slopes by age‐adjusted NfL residual group.

NfL z‐score group	Slope (ΔEDSS/visit)	95% CI	*p*‐value*
(A) EDSS progression slopes			
Low (−1 SD)	−0.016	[0.008, 0.046]	0.110
Mean (0 SD)	−0.032	[0.079, 0.016]	0.190
High (+1 SD)	0.053	[−0.002, 0.107]	0.048
Very High (+2 SD)	0.137	[0.053, 0.222]	0.002
(B) SSPROM progression slopes			
Low (−1 SD)	−0.195	[−0.701, 0.311]	0.448
Mean (0 SD)	−0.556	[−0.904, −0.207]	0.002
High (+1 SD)	−0.916	[−1.315, −0.517]	< 0.001
Very High (+2 SD)	−1.276	[−1.885, −0.668]	< 0.001
(C) 6MWT progression slopes			
Low (−1 SD)	−5.691	[−12.858, 1.476]	0.119
Mean (0 SD)	−6.250	[−11.371, −1.130]	0.170
High (+1 SD)	−6.810	[−12.313, −1.307]	0.015
Very High (+2 SD)	−7.369	[−15.346, 0.608]	0.007

*Note:* Table 3A−C presents estimated annual progression slopes for EDSS (A), SSPROM (B), and 6‐min walking test (6MWT) (C) derived from linear mixed‐effects models with random intercepts and slopes. Slopes represent the estimated mean change in outcome per visit (approximately one year). Models were adjusted for age and included a time × NfL residual interaction. *P‐values correspond to tests of whether the slope differs from zero within each NfL residual group.

Abbreviations: EDSS (Expanded Disability Status Scale), SSPROM (Severity Scoring system for Myelopathy), 6‐MWT: 6‐min walk test.

Pairwise comparisons between the slopes confirmed significant differences between mean, high, and very residual groups for EDSS (*p* < 0.001) and SSPROM after FDR correction (*p* = 0.026) (Table 4A–C). For SSPROM, the slope between the low residual group (−1 SD) and the mean group was also statistically significant (*p* = 0.026).

**TABLE 4:** Pairwise differences in slopes between NfL z‐score groups.

Comparison	ΔSlope	95% CI	*p* (FDR)
(A) EDSS Slope comparisons			
Low (−1 SD) − Mean (0 SD)	−0.016	[−0.151, −0.018]	0.170
Mean (0 SD) − High (+1 SD)	−0.084	[−0.151, −0.018]	< 0.001
Mean (0 SD) − Very High (+2 SD)	−0.169	[−0.302, −0.036]	< 0.001
(B) SSPROM Slope comparisons			
Low (−1 SD) − Mean (0 SD)	0.360	[−0.068, 0.789]	0.026
Mean (0 SD) − High (+1 SD)	0.360	[−0.068, 0.789]	0.026
Mean (0 SD) − Very High (+2 SD)	0.721	[−0.136, 1.578]	0.026
(C) 6MWT Slope comparisons			
Low (−1 SD) − Mean (0 SD)	0.559	[−4.613, 5.732]	0.774
Mean (0 SD) − High (+1 SD)	0.559	[−4.613, 5.732]	0.774
Mean (0 SD) − Very High (+2 SD)	1.119	[−9.227, 11.464]	0.774

*Note:* Table 4A–C present pairwise comparisons of annual progression slopes between age‐adjusted NfL residual groups for EDSS (A), SSPROM (B), and 6‐min walking test (6MWT) (C). ΔSlope represents the difference in estimated annual slope between the two groups being compared (slope of group A minus slope of group B). Positive ΔSlope values indicate faster worsening in the second group for EDSS, and faster worsening in the first group for SSPROM and 6MWT, consistent with scale directionality. Ninety‐five percent confidence intervals (95% CIs) are shown. *P*‐values were adjusted using false discovery rate (FDR) correction to account for multiple pairwise comparisons within each outcome.

### 3.7 Yearly Cumulative Clinical Change by Age‐Adjusted NfL Residual

Pairwise comparisons demonstrated higher age‐adjusted NfL residuals showing greater and earlier cumulative worsening of clinical outcomes. Participants in the Very High (+2 SD) group showed the most pronounced progression, with statistically significant deterioration evident from early follow‐up visits as early as V1–V0 for SSPROM, *p* < 0.001, and from V3–V0 and V6–V0 for EDSS and 6‐MWT, respectively. Significant changes in the high residual group (+1 SD) were seen for both EDSS, SSPROM, and 6‐MWT, albeit later (Supplementary Table 3A–C). The low residual group (−1 SD) didn't show any significant change in the clinical outcome measures, and the mean group showed only a significant change for SSPROM (Supplementary Table 3A–C).

**FIGURE 2:**
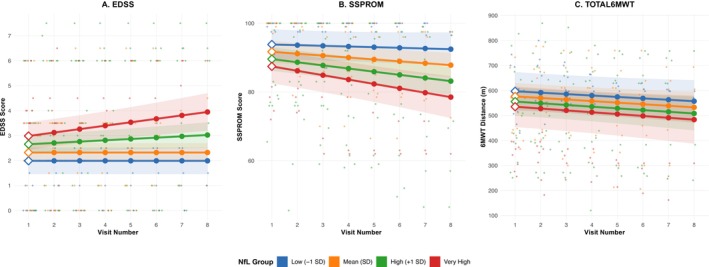
(A−C). Longitudinal trajectories of clinical outcomes by age‐adjusted NfL residual groups. Predicted longitudinal trajectories of clinical outcome measures stratified by age‐adjusted plasma neurofilament light (NfL) residual groups, expressed in standard deviation (SD) units relative to the cohort. Age‐adjusted NfL residuals were derived as standardized residuals from baseline log‐transformed NfL regressed on age and categorized into four groups: Low (−1 SD; *n* = 18), Mean (0 SD; *n* = 26), High (+1 SD; *n* = 27), and Very High (+2 SD; *n* = 16). Predictions were obtained from linear mixed‐effects models including time (visit number) as a continuous variable and random intercepts and slopes for participants. Solid lines represent model‐estimated mean trajectories, shaded bands indicate 95% confidence intervals, and individual dots represent observed measurements at each visit. Higher age‐adjusted NfL residuals were associated with steeper EDSS progression and greater decline in SSPROM over time, whereas no clear between‐group differences were observed for 6‐MWT trajectories. Abbreviations: (A) Expanded Disability Status Scale (EDSS), (B) Severity Scoring System for Myelopathy (SSPROM), (C) 6‐Minute Walking Test (6‐MWT). NfL, neurofilament light; SD, standard deviation.

See Figure 2A–C for the course of clinical change of the age‐adjusted NfL residual groups.

### 3.8 GFAP Change Over Time

There was a statistically significant effect of time on GFAP plasma levels (*p* = 0.031) (Supplementary Figure 2). Estimated marginal means ranged from 73.0 pg/mL (Baseline, SE = 5.40, 95% CI [55.5, 90.5]) to 103.5 (Visit 7, SE = 13.20, 95% CI [70.8–136.2]). Pairwise comparisons, adjusted for multiple comparisons with FDR, revealed a significant difference between V7−V0 (*p* = 0.017) (Supplementary Table 1).

### 3.9 Longitudinal Predictive Value of GFAP

**FIGURE 3:**
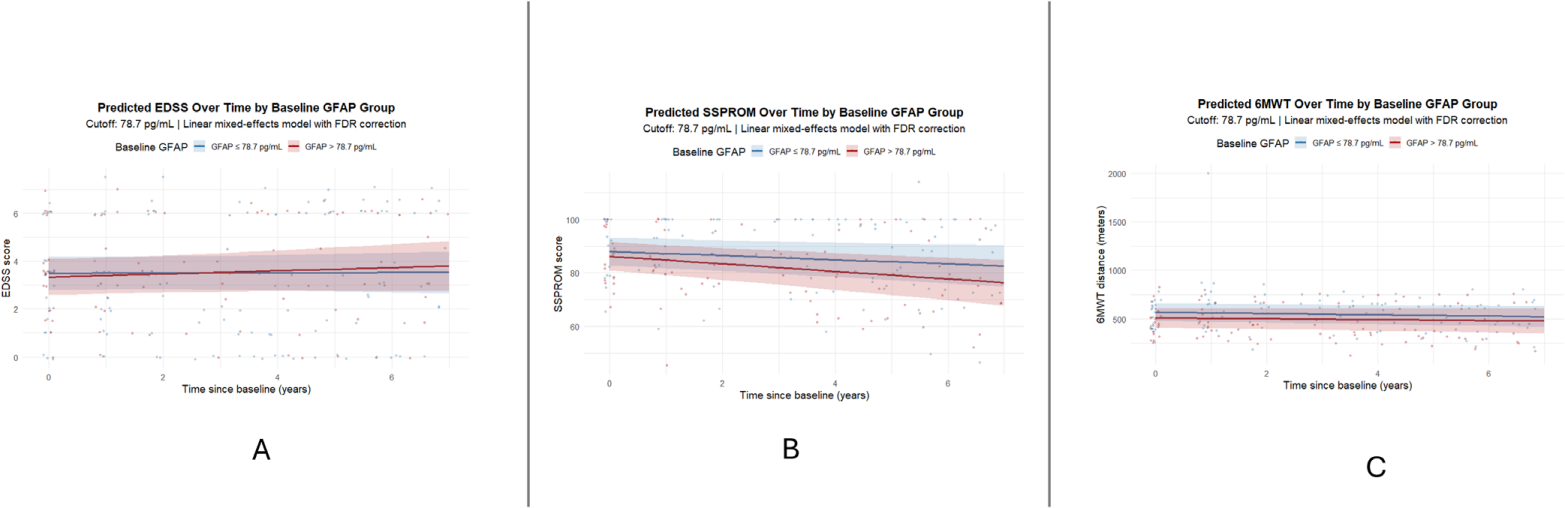
(A−C) Longitudinal Trajectories of clinical outcomes stratified by baseline plasma GFAP (78.7 pg/mL cut‐off).Predicted longitudinal trajectories of clinical outcome measures stratified by baseline plasma neurofilament light (NfL) concentration using a cutoff of 78.7 pg/mL. Participants were classified as having low GFAP (< 78.7 pg/mL) (*n* = 40) or high GFAP (≥ 78.7 pg/mL) (*n* = 12) at baseline. Predictions were obtained from linear mixed‐effects models including time as a continuous variable and random intercepts and slopes for participants, adjusted for age. Solid lines represent model‐estimated mean trajectories, shaded bands indicate 95% confidence intervals, and individual dots represent observed measurements at each visit. Abbreviations: (A) Expanded Disability Status Scale (EDSS), (B) Severity Scoring System for Myelopathy (SSPROM), (C) 6‐Minute Walking Test (6‐MWT). Abbreviations: NfL: Neurofilament Light Chain; pg/mL: Picograms per milliliters; EDSS: Expanded Disability Status Scale; SSPROM: Severity Scoring System for Myelopathy; 6‐MWT: 6‐min walk test.

For EDSS, no significant changes were observed over time in either group (Figure 3A). There was no significant difference in the slope between the two subgroups (β = −0.060, *p* = 0.391) (Table 12). See Supplementary Table 4A for a detailed overview of the mean estimated EDSS change per year.

For SSPROM (Figure 3B), a significant decline over time was observed in both the GFAP ≥ 78.7 pg/mL group and the GFAP < 78.7 pg/mL group. In the GFAP ≥ 78.7 pg/mL group, pairwise comparisons showed a change of −1.41 at V1–V0 (95% CI: −2.46 to −0.37, effect size 0.65) progressing to −9.90 by V7–V0 (*p* < 0.001). The < 78.7 pg/mL group also showed a significant decline (*p* = 0.023) from −0.79 to −5.56 over the same period (Supplementary Table 4B). The slope of decline between the groups did not differ statistically significantly (β = +0.62, *p* = 0.176) (Table 5).

**TABLE 5:** Comparison of Annual Rates of Change Across Outcomes GFAP < 78.7 pg/mL vs. ≥ 78.7 pg/mL.

Outcome	Between‐Group Slope Difference Δ [95% CI]	*p*‐ value
EDSS	Δ = −0.060 [−0.202 to 0.081]	0.391
SSPROM	Δ = +0.620 [−0.286 to 1.558]	0.176
6MWT (m)	Δ = 0.997 [−12.544 to 8.230]	0.997

*Note:* Δ (Between‐Group Slope Difference) is defined as High‐Low (compared to Time × GFAP_group Under 78.7 interaction term).

Abbreviations: EDSS (Expanded Disability Status Scale), SSPROM (Severity Scoring system for Myelopathy), 6‐MWT: 6‐min walk test.

* *p*‐value is for the Time × NfL group interaction.

For the 6‐MWT, a non‐significant decline over time was seen in both the ≥ 78.7 pg/mL group (V7–V0, p = 0.318) and the < 78.7 pg/mL group (V7–V0, p = 0.233). The time × GFAP interaction was not significant (β = +0.016, *p* = 0.997), indicating no difference in decline rates between groups (Table 12). Figure 3C shows the course of 6‐MWT outcomes for both subgroups over time. Detailed results can be found in Supplementary Table 4C.

## 4 Discussion

In this study, an extension of the cohort investigated by van Ballegoij et al. [17], we describe the prognostic value of NfL and GFAP regarding the progression of spinal cord disease in male X‐ALD patients over a seven‐year clinical follow‐up. Longitudinal analysis of plasma neurofilament light chain (NfL) and glial fibrillary acidic protein (GFAP) levels over a seven‐year period revealed no statistically significant effect of time on serum NfL levels (*p* = 0.111), though pairwise comparisons revealed a statistically significant increase between V6 and baseline visit (mean change = 5.9 pg/mL, *p* = 0.003) and between baseline and V7 (mean change = 5.4 pg/mL, *p* = 0.002). In contrast, GFAP levels demonstrated a statistically significant overall increase over time (*p* = 0.031); however, pairwise comparisons revealed only significant differences between baseline and V7. These findings suggest that neither biomarker exhibits temporal changes over time.

However, stratification by baseline biomarker levels provided deeper insights. Patients with higher baseline NfL (≥ 15.7 pg/mL) exhibited significantly greater disease progression, as evidenced by increased EDSS scores (*p* < 0.001) and a steeper disability progression slope (β = −0.27, *p* < 0.001) compared to those with lower NfL levels. Although the ≥ 15.7 pg/mL NfL group and < 15.7 pg/mL NfL group declined both significantly (*p* = 0.001 and *p* = 0.008, respectively), the slope‐difference wasn't significantly different (*p* = 0.060). Like EDSS, 6‐MWT outcomes showed a decline in only the ≥ 15.7 pg/mL NfL subgroup; however, there were no significant differences in the slope of decline.

In contrast, GFAP stratification showed limited prognostic utility, with only the ≥ 78.7 pg/mL group demonstrating a significant decline in SSPROM scores in both subgroups (*p* < 0.001 and *p* = 0.0023), and no significant changes in EDSS or 6‐MWT outcomes. The lack of significant differences in progression slopes between GFAP subgroups further underscores its limited prognostic value. Our results strongly support the use of serum NfL as a clinically relevant biomarker for disease severity and progression, while GFAP shows limited prognostic value.

### 4.1 NFL and GFAP Baseline Assessments After Stratification

Participants with higher NfL at baseline showed a more severe disability, indicated by a higher EDSS, lower SSPROM, and lower distances at the 6‐MWT. Our findings corroborate Ballegoij et al. [17], who showed significant correlations between NfL levels and clinical parameters for disease severity, supporting NfL's role as a marker of axonal degeneration. Notably, patients in the NfL ≥ 15 pg/mL subgroup were significantly older, a confounding factor also noted by van Ballegoij et al. [17], as aging and myeloneuropathy progression both contribute to elevated NfL. Even after adjusting for age, NfL remained a significant predictor of disease severity.

In contrast, no significant differences were observed in the EDSS, SSPROM, or 6‐MWT scores between GFAP subgroups. This lack of correlation at baseline suggests that while GFAP may reflect astrocytic activation or gliosis, it may not directly align with functional impairment or disease severity in X‐ALD at the individual patient level.

Our findings build on a growing body of literature emphasizing NfL as a sensitive marker of axonal damage in X‐ALD [18, 22]. In CALD, for example, Weinhofer et al. [22], reported significantly elevated NfL in myeloneuropathy patients who later developed CALD, with levels reaching those in amyotrophic lateral sclerosis (ALS) during active CALD. Lund's recent study in the pediatric population found a > 50% NfL increase from baseline to be a robust indicator of cerebral lesion development, particularly in patients who showed gadolinium enhancement [18]. Our findings in adult myeloneuropathies complement these studies, where NfL tracks chronic, slower degeneration in addition to its ability to reflect acute axonal damage in CALD.

### 4.2 Strength and Limitations

Several limitations should be considered when interpreting these findings. First, plasma NfL concentrations were analyzed primarily using absolute values rather than normative, population‐based z‐scores. Although age‐adjusted NfL z‐scores derived from large healthy control cohorts have been shown to improve prognostic performance in disorders such as multiple sclerosis [23], their biological relevance in ALD is less clear. ALD is characterized by a slow, continuous neurodegenerative process rather than episodic inflammatory activity. In this setting, absolute NfL concentrations may more directly capture cumulative axonal injury and overall disease burden.

A key challenge in interpreting baseline NfL levels in X‐ALD is the strong association between NfL and age, which is well documented in healthy individuals and neurological populations. Large normative studies have demonstrated a gradual increase in plasma NfL across the lifespan, with acceleration in older age decades [20, 21]. In our cohort, patients with high baseline NfL were significantly older than those with lower NfL levels, raising the concern that NfL might simply reflect chronological aging rather than disease‐specific neurodegeneration.

To address this, we performed sensitivity analyses using an internally derived, age‐adjusted NfL metric based on standardized residuals from baseline log(NfL) regressed on age. This approach does not rely on external healthy reference values but instead captures NfL elevations beyond those expected for age within the X‐ALD cohort itself. Importantly, the association between higher age‐adjusted NfL and faster clinical progression—most notably for EDSS—persisted in these analyses, supporting the interpretation that NfL reflects disease‐related axonal injury rather than age alone.

Additionally, the results of our analyses are limited by the limited value of the sensitivity of the used outcome measures [1, 5, 6, 7]. As the myeloneuropathy is slowly progressing, there is a lag between axonal degeneration and disability until a critical threshold is reached, which is also reflected in the clinical scales. Nonetheless, significant changes are seen in the most sensitive clinical outcome scale, the EDSS [6, 7].

Stratification based on EDSS levels has previously been shown to be a valuable tool in the prediction of disease course. However, the dynamic nature of NfL contrasts with clinical measures like the EDSS, which capture accumulated disability. This makes the NfL particularly valuable for monitoring presymptomatic patients, as was earlier suggested in the literature, where elevated NfL may precede symptoms.

## 5 Conclusion

Given the significant effect sizes and early separation in disease trajectories in this longest biochemical prospective follow‐up in adult X‐ALD males with myeloneuropathy, serum NfL should be considered for integration into clinical trial protocols as both a stratification tool and a surrogate outcome measure.

In contrast, GFAP's limited predictive capacity suggests that it may serve better as a complementary biomarker than a standalone marker of disease progression.

## Author Contributions

Eda G. Kabak: Data curation, Formal analysis, Investigation, Writing – original draft. Marije M.C. Voermans: Data curation, Project administration. Hans Heijst: Sample analyses, review. Charlotte E. Teunissen: Methodology, Writing – review and editing. Marc Engelen: Conceptualization, Supervision, Funding acquisition, Writing – review and editing. Guarantor author: Marc Engelen.

## Funding

This work was supported by the Nederlandse Organisatie voor Wetenschappelijk Onderzoek (016.196.310).

## Conflicts of Interest

C.E. Teunissen: C.E.T. has research contracts with Acumen, ADx Neurosciences, AC‐Immune, Alamar, Aribio, Axon Neurosciences, Beckman‐Coulter, BioConnect, Bioorchestra, Brainstorm Therapeutics, C2N diagnostics, Celgene, Cognition Therapeutics, EIP Pharma, Eisai, Eli Lilly, Fujirebio, Instant Nano Biosensors, Merck, Muna, Nitrase Therapeutics, Novo Nordisk, Olink, PeopleBio, Quanterix, Roche, Sysmex, Toyama, Vaccinex, Vivoryon.

She is editor in chief of Alzheimer Research and Therapy, and serves on the editorial boards of Molecular Neurodegeneration, Alzheimer's & Dementia, Neurology: Neuroimmunology & Neuroinflammation, Medidact Neurologie/Springer, and is a committee member to define guidelines for Cognitive disturbances, and one for acute Neurology in the Netherlands. She has consultancy/speaker contracts for Aribio, Biogen, Beckman‐Coulter, Cognition Therapeutics, Danaher, Eisai, Eli Lilly, Janssen, Merck, Neurogen Biomarking, Nordic Biosciences, Novo Nordisk, Novartis, Olink, Quanterix, Roche, Sanofi, and Veravas. M. Engelen is co‐PI for Spur Therapeutics (CYGNET and PROPEL trials) and was co‐PI for Minoryx (ADVANCE trial). He has received research support from Minoryx, Autobahn Therapeutics, BlueBirdBio, and SwanBio (now Spur Therapeutics); consultation fees from Minoryx, BlueBirdBio, Autobahn Therapeutics, and Poxel. Other authors report no further disclosures.

## Supporting information


**Supplementary Table 1:** Combined mean estimated changes in NfL and GFAP during follow‐up (vs baseline).
**Supplementary Table 2A:** Mean estimated EDSS change stratified by NfL subgroup.
**Supplementary Table 2B:** Estimated SSPROM change stratified by NfL subgroup.
**Supplementary Table 2C:** Mean estimated change 6‐MWT stratified by NfL subgroup.
**Supplementary Table 3:** Model‐estimated cumulative change from baseline by age‐adjusted NfL residual group.
**Supplementary Table 4A:** Mean estimated EDSS change stratified by GFAP subgroup.
**Supplementary Table 4B:** Mean estimated SSPROM change stratified by GFAP subgroup.
**Supplementary Table 4C:** Mean estimated change 6‐MWT stratified by GFAP subgroup.
**Supplementary Figure 1:** Title: sNfL trajectory over time.
Predicted plasma NfL (sNfL) concentrations across study visits (baseline to year 7) were estimated using a linear mixed‐effects model with visit treated as a continuous variable and random intercepts and slopes for participants. The solid line represents the model‐estimated mean NfL trajectory, and the shaded area indicates the 95% confidence interval. No statistically significant linear change in plasma NfL over time was observed (p = 0.111).
**Supplementary Figure 2:** Title: sGFAP trajectory over time.
Predicted plasma GFAP (sGFAP) concentrations across study visits (baseline to year 7) were estimated using a linear mixed‐effects model with visit treated as a continuous variable and random intercepts and slopes for participants. The solid line represents the model‐estimated mean GFAP trajectory, and the shaded area indicates the 95% confidence interval. A statistically significant increase in plasma GFAP over time was observed (linear visit effect *p* = 0.0087).

## Data Availability

The data that support the findings of this study are available from the corresponding author upon reasonable request.
