# Supplementary material for: Prognostic Value of Neurofilament Light Chain and Glial Fibrillary Acidic Protein in ALD‐Related Myelopathy
Source: Ann Clin Transl Neurol. 2026 Apr 8;13(10):2114–23. doi: 10.1002/acn3.70386 (PMC13394911; doi:10.1002/acn3.70386)
Supplement: Supplementary file 1 [file ACN3-13-2114-s001.docx]

# Supplementary tables

**Supplementary Table 1: Combined mean estimated changes in NfL and GFAP during follow-up (vs baseline)**

| Period (FUY – baseline) | NfL change (pg/mL) | SE | p (FDR)* | GFAP change (pg/mL) | SE | p* |
| --- | --- | --- | --- | --- | --- | --- |
| V1-V0 | 0.272 | 1.20 | 0.931 | 4.221 | 5.40 | 0.291 |
| V2-V0 | 0.122 | 1.36 | 0.931 | 4.757 | 5.55 | 0.291 |
| V3-V0 | 2.085 | 1.41 | 0.211 | 14.268 | 6.38 | 0.300 |
| V4-V0 | 2.879 | 1.52 | 0.119 | 20.872 | 7.96 | 0.100 |
| V5-V0 | 3.443 | 1.46 | 0.690 | 19.760 | 5.6 | 0.090 |
| V6-V0 | 5.953 | 1.58 | 0.003 | 18.508 | 9.63 | 0.060 |
| V7-V0 | 5.489 | 1.80 | 0.002 | 18.158 | 13.20 | 0.017 |

Periods are expressed as Follow-up Year – baseline. Values are presented as estimated mean change from baseline (units: pg/mL).

p (FDR)*: p-values are false discovery rate (FDR) adjusted across follow-up timepoints within each biomarker.

V0 Estimated marginal means for baseline
V1 Estimated marginal means for follow-up year 1

V2 Estimated marginal means for follow-up year 2

V3 Estimated marginal means for follow-up year 3

V4 Estimated marginal means for follow-up year 4

V5 Estimated marginal means for follow-up year 5

V6 Estimated marginal means for follow-up year 6

V7 Estimated marginal means for follow-up year 7

**Supplementary table 2A: Mean estimated EDSS change stratified by NfL subgroup**

| NfL-subgroup | Period | Estimated Mean Change | SE | p-value* | 95% CI: Lower bound | 95% CI upper bound | Effect size |
| --- | --- | --- | --- | --- | --- | --- | --- |
| ≥15.7 pg/ml | V1-V0 | 0.24 | 0.06 | 0.002 | 0.07 | 0.42 | 0.40 |
|  | V2-V0 | 0.49 | 0.13 | 0.002 | 0.13 | 0.85 | 0.40 |
|  | V3-V0 | 0.73 | 0.19 | <0.001 | 0.20 | 1.27 | 0.45 |
|  | V4-V0 | 0.97 | 0.26 | <0.001 | 0.26 | 1.69 | 0.45 |
|  | V5-V0 | 1.23 | 0.32 | <0.001 | 0.32 | 2.12 | 0.45 |
|  | V6-V0 | 1.46 | 0.38 | <0.001 | 0.38 | 2.54 | 0.45 |
|  | V7-V0 | 1.70 | 0.45 | <0.001 | 0.44 | 2.96 | 0.45 |
| < 15.7 pg/ml | V1-V0 | -0.03 | 0.03 | 0.622 | -0.13 | 0.07 | 0.09 |
|  | V2-V0 | -0.06 | 0.07 | 0.572 | -0.26 | 0.13 | 0.09 |
|  | V3-V0 | -0.09 | 0.10 | 0.522 | -0.39 | 0.20 | 0.09 |
|  | V4-V0 | -0.13 | 0.13 | 0.492 | -0.51 | 0.26 | 0.09 |
|  | V5-V0 | -0.16 | 0.17 | 0.422 | -0.64 | 0.33 | 0.10 |
|  | V6-V0 | -0.19 | 0.21 | 0.392 | -0.77 | 0.39 | 0.10 |
|  | V7-V0 | -0.22 | 0.24 | 0.322 | -0.90 | 0.46 | 0.11 |

Data are estimated mean changes (95% CI) from linear mixed-effects models for high (≥15.7 pg/mL) vs. low (<15.7 pg/mL) baseline NfL groups.
 *p*-values are FDR-corrected. Effect sizes (Cohen's d) are shown for the overall change.

**Abbreviations:** EDSS, Expanded Disability Status Scale; FDR, false discovery rate; NfL, neurofilament light chain; SSPROM, Severity Scoring System for Progressive Myelopathy; 6MWT, Six-Minute Walk Test.

V0 Estimated marginal means for baseline; V1 Estimated marginal means for follow-up year 1; V2 Estimated marginal means for follow-up year 2; V3 Estimated marginal means for follow-up year 3; V4 Estimated marginal means for follow-up year 4; V5 Estimated marginal means for follow-up year 5; V6 Estimated marginal means for follow-up year 6; V7 Estimated marginal means for follow-up year 7

Effect size: Partial eta squared for the repeated measures analyses of variance

**Supplementary table 2B: Estimated SSPROM change stratified by NfL subgroup**

| NfL-subgroup | Period | Estimated Mean Change | SE | p-value* | 95% CI Lower bound | 95% CI upper bound | Effect size |
| --- | --- | --- | --- | --- | --- | --- | --- |
| ≥15.7 pg/mL | V1-V0 | -1.68 | 0.46 | 0.001 | -2.99 | -0.37 | 0.40 |
|  | V2-V0 | -3.35 | 0.92 | 0.001 | -5.97 | -0.73 | 0.40 |
|  | V3-V0 | -5.03 | 1.38 | 0.001 | -8.96 | -1.10 | 0.43 |
|  | V4-V0 | -6.70 | 1.84 | <0.001 | -11.94 | -1.46 | 0.43 |
|  | V5-V0 | -8.38 | 2.30 | <0.001 | -14.93 | -1.83 | 0.43 |
|  | V6-V0 | -10.05 | 2.76 | <0.001 | -17.91 | -2.20 | 0.43 |
|  | V7-V0 | -11.73 | 3.22 | <0.001 | -20.90 | -2.56 | 0.43 |
| < 15.7 pg/mL | V1-V0 | -0.72 | 0.25 | 0.008 | -1.43 | -0.01 | 0.30 |
|  | V2-V0 | -1.44 | 0.50 | 0.008 | -2.86 | -0.02 | 0.30 |
|  | V3-V0 | -2.16 | 0.75 | 0.007 | -4.28 | -0.03 | 0.32 |
|  | V4-V0 | -2.88 | 1.00 | 0.007 | -5.71 | -0.04 | 0.32 |
|  | V5-V0 | -3.59 | 1.25 | 0.006 | -7.14 | -0.05 | 0.34 |
|  | V6-V0 | -4.31 | 1.50 | 0.006 | -8.57 | -0.06 | 0.34 |
|  | V7-V0 | -5.03 | 1.76 | 0.006 | -9.99 | -0.07 | 0.34 |

Data are estimated mean changes (95% CI) from linear mixed-effects models for high (≥15.7 pg/mL) vs. low (<15.7 pg/mL) baseline NfL groups.
 *p*-values are FDR-corrected. Effect sizes (Cohen's d) are shown for the overall change.

**Abbreviations:** EDSS, Expanded Disability Status Scale; FDR, false discovery rate; NfL, neurofilament light chain; SSPROM, Severity Scoring System for Progressive Myelopathy; 6MWT, Six-Minute Walk Test.

V0 Estimated marginal means for baseline; V1 Estimated marginal means for follow-up year 1; V2 Estimated marginal means for follow-up year 2; V3 Estimated marginal means for follow-up year 3; V4 Estimated marginal means for follow-up year 4; V5 Estimated marginal means for follow-up year 5; V6 Estimated marginal means for follow-up year 6; V7 Estimated marginal means for follow-up year 7

Effect size: Partial eta squared for the repeated measures analyses of variance

**Supplementary Table 2C: Mean estimated change 6-MWT stratified by NfL subgroup**

| NfL-subgroup | Period | Estimated Mean Change | SE | p-value* | 95% CI: Lower bound | 95% CI upper bound | Effect size |
| --- | --- | --- | --- | --- | --- | --- | --- |
| ≥15.7 pg/ml | V1-V0 | -11.58 | 5.29 | 0.300 | -26.49 | 3.33 | 0.14 |
|  | V2-V0 | -23.16 | 10.58 | 0.195 | -52.98 | 6.66 | 0.14 |
|  | V3-V0 | -34.74 | 15.87 | 0.100 | -79.47 | 9.99 | 0.15 |
|  | V4-V0 | -46.31 | 21.16 | 0.080 | -105.96 | 13.33 | 0.18 |
|  | V5-V0 | -57.89 | 26.45 | 0.051 | -132.44 | 16.66 | 0.20 |
|  | V6-V0 | -69.47 | 31.74 | 0.049 | -158.93 | 19.99 | 0.24 |
|  | V7-V0 | -81.05 | 37.03 | 0.038 | -185.42 | 23.32 | 0.26 |
| < 15.7 pg/ml | V1-V0 | -6.51 | 3.21 | 0.425 | -15.45 | 2.42 | 0.07 |
|  | V2-V0 | -13.03 | 6.43 | 0.415 | -30.90 | 4.85 | 0.10 |
|  | V3-V0 | -19.54 | 9.64 | 0.375 | -46.35 | 7.27 | 0.12 |
|  | V4-V0 | -26.05 | 12.85 | 0.295 | -61.80 | 9.69 | 0.17 |
|  | V5-V0 | -32.57 | 16.07 | 0.205 | -77.25 | 12.12 | 0.19 |
|  | V6-V0 | -39.08 | 19.28 | 0.195 | -92.70 | 14.54 | 0.20 |
|  | V7-V0 | -45.59 | 22.50 | 0.095 | -108.15 | 16.97 | 0.24 |

Data are estimated mean changes (95% CI) from linear mixed-effects models for high (≥15.7 pg/mL) vs. low (<15.7 pg/mL) baseline NfL groups.
 *p*-values are FDR-corrected. Effect sizes (Cohen's d) are shown for the overall change.

**Abbreviations:** EDSS, Expanded Disability Status Scale; FDR, false discovery rate; NfL, neurofilament light chain; SSPROM, Severity Scoring System for Progressive Myelopathy; 6MWT, Six-Minute Walk Test.

V0 Estimated marginal means for baseline; V1 Estimated marginal means for follow-up year 1; V2 Estimated marginal means for follow-up year 2; V3 Estimated marginal means for follow-up year 3; V4 Estimated marginal means for follow-up year 4; V5 Estimated marginal means for follow-up year 5; V6 Estimated marginal means for follow-up year 6; V7 Estimated marginal means for follow-up year 7

Effect size: Partial eta squared for the repeated measures analyses of variance

**Supplementary Table 3. Model-estimated cumulative change from baseline by age-adjusted NfL residual group**

Table 3A. Cumulative EDSS change from baseline

| NfL z-score group | Period | Cumulative ΔEDSS | 95% CI | p-value | Effect size (Cohen’s d) |
| --- | --- | --- | --- | --- | --- |
| Low (−1 SD) | V1-V0 | −0.02 | [−0.09, −0.01] | 0.410 | −0.17 |
| Low (−1 SD) | V2-V0 | −0.04 | [−0.37, −0.09] | 0.385 | −0.34 |
| Low (−1 SD) | V3-V0 | −0.06 | [−0.56, −0.14] | 0.345 | −0.51 |
| Low (−1 SD) | V4-V0 | −0.07 | [−0.75, −0.18] | 0.295 | −0.68 |
| Low (−1 SD) | V5-V0 | −0.08 | [−0.93, −0.23] | 0.235 | −0.85 |
| Low (−1 SD) | V6-V0 | −0.10 | [−1.12, −0.27] | 0.165 | −1.02 |
| Low (−1 SD) | V7-V0 | −0.12 | [−1.30, −0.32] | 0.110 | −1.19 |
| Mean (0 SD) | V1-V0 | −0.03 | [−0.08, 0.02] | 0.410 | −0.05 |
| Mean (0 SD) | V2-V0 | −0.06 | [−0.16, 0.03] | 0.375 | −0.09 |
| Mean (0 SD) | V3-V0 | −0.09 | [−0.24, 0.05] | 0.330 | −0.14 |
| Mean (0 SD) | V4-V0 | −0.13 | [−0.32, 0.06] | 0.280 | −0.19 |
| Mean (0 SD) | V5-V0 | −0.16 | [−0.39, 0.08] | 0.225 | −0.23 |
| Mean (0 SD) | V6-V0 | −0.19 | [−0.47, 0.09] | 0.175 | −0.28 |
| Mean (0 SD) | V7-V0 | −0.22 | [−0.55, 0.11] | 0.190 | −0.32 |
| High (+1 SD) | V1-V0 | 0.05 | [−0.00, 0.11] | 0.310 | 0.08 |
| High (+1 SD) | V2-V0 | 0.11 | [−0.00, 0.21] | 0.260 | 0.16 |
| High (+1 SD) | V3-V0 | 0.16 | [−0.01, 0.32] | 0.195 | 0.23 |
| High (+1 SD) | V4-V0 | 0.21 | [−0.01, 0.43] | 0.050 | 0.31 |
| High (+1 SD) | V5-V0 | 0.26 | [−0.01, 0.54] | 0.040 | 0.39 |
| High (+1 SD) | V6-V0 | 0.32 | [−0.01, 0.64] | 0.035 | 0.47 |
| High (+1 SD) | V7-V0 | 0.37 | [−0.01, 0.75] | 0.028 | 0.54 |
| Very High (+2 SD) | V1-V0 | 0.14 | [0.05, 0.22] | 0.380 | 0.20 |
| Very High (+2 SD) | V2-V0 | 0.27 | [0.11, 0.44] | 0.120 | 0.40 |
| Very High (+2 SD) | V3-V0 | 0.41 | [0.16, 0.67] | 0.050 | 0.60 |
| Very High (+2 SD) | V4-V0 | 0.55 | [0.21, 0.89] | 0.045 | 0.81 |
| Very High (+2 SD) | V5-V0 | 0.69 | [0.26, 1.11] | 0.038 | 1.01 |
| Very High (+2 SD) | V6-V0 | 0.82 | [0.32, 1.33] | 0.018 | 1.21 |
| Very High (+2 SD) | V7-V0 | 0.96 | [0.37, 1.55] | 0.002 | 1.41 |

Table 3B. Cumulative SSPROM change from baseline

| NfL z-score group | Period | Cumulative ΔSSPROM | 95% CI | p-value | Effect size (Cohen’s d) |
| --- | --- | --- | --- | --- | --- |
| Low (−1 SD) | V1-V0 | −0.20 | [−0.70, 0.31] | 0.480 | −0.04 |
| Low (−1 SD) | V2-V0 | −0.39 | [−1.40, 0.62] | 0.465 | −0.08 |
| Low (−1 SD) | V3-V0 | −0.59 | [−2.10, 0.93] | 0.440 | −0.13 |
| Low (−1 SD) | V4-V0 | −0.78 | [−2.80, 1.24] | 0.405 | −0.17 |
| Low (−1 SD) | V5-V0 | −0.98 | [−3.51, 1.55] | 0.360 | −0.21 |
| Low (−1 SD) | V6-V0 | −1.17 | [−4.21, 1.87] | 0.300 | −0.25 |
| Low (−1 SD) | V7-V0 | −1.37 | [−4.91, 2.18] | 0.448 | −0.29 |
| Mean (0 SD) | V1-V0 | −0.56 | [−0.90, −0.21] | 0.380 | −0.12 |
| Mean (0 SD) | V2-V0 | −1.11 | [−1.81, −0.41] | 0.280 | −0.24 |
| Mean (0 SD) | V3-V0 | −1.67 | [−2.71, −0.62] | 0.190 | −0.36 |
| Mean (0 SD) | V4-V0 | −2.22 | [−3.61, −0.83] | 0.120 | −0.48 |
| Mean (0 SD) | V5-V0 | −2.78 | [−4.52, −1.04] | 0.070 | −0.60 |
| Mean (0 SD) | V6-V0 | −3.33 | [−5.42, −1.24] | 0.035 | −0.72 |
| Mean (0 SD) | V7-V0 | −3.89 | [−6.33, −1.45] | 0.002 | −0.84 |
| High (+1 SD) | V1-V0 | −0.92 | [−1.31, −0.52] | 0.075 | −0.20 |
| High (+1 SD) | V2-V0 | −1.83 | [−2.63, −1.03] | 0.040 | −0.39 |
| High (+1 SD) | V3-V0 | −2.75 | [−3.94, −1.55] | 0.018 | −0.59 |
| High (+1 SD) | V4-V0 | −3.66 | [−5.26, −2.07] | <0.001 | −0.79 |
| High (+1 SD) | V5-V0 | −4.58 | [−6.57, −2.59] | <0.001 | −0.99 |
| High (+1 SD) | V6-V0 | −5.50 | [−7.89, −3.10] | <0.001 | −1.18 |
| High (+1 SD) | V7-V0 | −6.41 | [−9.20, −3.62] | <0.001 | −1.38 |
| Very High (+2 SD) | V1-V0 | −1.28 | [−1.88, −0.67] | <0.001 | −0.27 |
| Very High (+2 SD) | V2-V0 | −2.55 | [−3.77, −1.34] | <0.001 | −0.55 |
| Very High (+2 SD) | V3-V0 | −3.83 | [−5.65, −2.00] | <0.001 | −0.82 |
| Very High (+2 SD) | V4-V0 | −5.11 | [−7.54, −2.67] | <0.001 | −1.10 |
| Very High (+2 SD) | V5-V0 | −6.38 | [−9.42, −3.34] | <0.001 | −1.37 |
| Very High (+2 SD) | V6-V0 | −7.66 | [−11.31, −4.01] | <0.001 | −1.65 |
| Very High (+2 SD) | V7-V0 | −8.94 | [−13.19, −4.68] | <0.001 | −1.92 |

Table 3C. Cumulative 6MWT change from baseline

| NfL z-score group | Period | Cumulative Δ6MWT (m) | 95% CI | p-value | Effect size (Cohen’s d) |
| --- | --- | --- | --- | --- | --- |
| Low (−1 SD) | V1-V0 | −5.69 | [−12.86, 1.48] | 0.420 | −0.10 |
| Low (−1 SD) | V2-V0 | −11.38 | [−25.72, 2.95] | 0.395 | −0.21 |
| Low (−1 SD) | V3-V0 | −17.07 | [−38.57, 4.43] | 0.355 | −0.31 |
| Low (−1 SD) | V4-V0 | −22.76 | [−51.43, 5.90] | 0.305 | −0.42 |
| Low (−1 SD) | V5-V0 | −28.46 | [−64.29, 7.38] | 0.245 | −0.52 |
| Low (−1 SD) | V6-V0 | −34.15 | [−77.15, 8.85] | 0.175 | −0.63 |
| Low (−1 SD) | V7-V0 | −39.84 | [−90.01, 10.33] | 0.119 | −0.73 |
| Mean (0 SD) | V1-V0 | −6.25 | [−11.37, −1.13] | 0.410 | −0.11 |
| Mean (0 SD) | V2-V0 | −12.50 | [−22.74, −2.26] | 0.370 | −0.23 |
| Mean (0 SD) | V3-V0 | −18.75 | [−34.11, −3.39] | 0.320 | −0.34 |
| Mean (0 SD) | V4-V0 | −25.00 | [−45.48, −4.52] | 0.265 | −0.46 |
| Mean (0 SD) | V5-V0 | −31.25 | [−56.85, −5.65] | 0.210 | −0.57 |
| Mean (0 SD) | V6-V0 | −37.50 | [−68.23, −6.78] | 0.160 | −0.69 |
| Mean (0 SD) | V7-V0 | −43.75 | [−79.60, −7.91] | 0.170 | −0.80 |
| High (+1 SD) | V1-V0 | −6.81 | [−12.31, −1.31] | 0.380 | −0.13 |
| High (+1 SD) | V2-V0 | −13.62 | [−24.63, −2.61] | 0.280 | −0.25 |
| High (+1 SD) | V3-V0 | −20.43 | [−36.94, −3.92] | 0.190 | −0.38 |
| High (+1 SD) | V4-V0 | −27.24 | [−49.25, −5.23] | 0.120 | −0.50 |
| High (+1 SD) | V5-V0 | −34.05 | [−61.57, −6.53] | 0.070 | −0.63 |
| High (+1 SD) | V6-V0 | −40.86 | [−73.88, −7.84] | 0.035 | −0.75 |
| High (+1 SD) | V7-V0 | −47.67 | [−86.19, −9.15] | 0.015 | −0.88 |
| Very High (+2 SD) | V1-V0 | −7.37 | [−15.35, 0.61] | 0.360 | −0.14 |
| Very High (+2 SD) | V2-V0 | −14.74 | [−30.69, 1.22] | 0.250 | −0.27 |
| Very High (+2 SD) | V3-V0 | −22.11 | [−46.04, 1.82] | 0.160 | −0.41 |
| Very High (+2 SD) | V4-V0 | −29.48 | [−61.38, 2.43] | 0.095 | −0.54 |
| Very High (+2 SD) | V5-V0 | −36.85 | [−76.73, 3.04] | 0.052 | −0.68 |
| Very High (+2 SD) | V6-V0 | −44.21 | [−92.08, 3.65] | 0.028 | −0.81 |
| Very High (+2 SD) | V7-V0 | −51.58 | [−107.42, 4.25] | 0.070 | −0.95 |

Tables **3A–C** present model-estimated cumulative changes from baseline in EDSS (A), SSPROM (B), and 6-minute walking test (6MWT) (C) across follow-up visits. Estimates were derived from linear mixed-effects models with random intercepts and slopes, including fixed effects for time, age-adjusted NfL residual group, age, and their interaction.

**Abbreviations:** EDSS (Expanded Disability Status Scale), SSPROM (Severity Scoring system for Myelopathy), 6-MWT: 6-minutes walk test.

SD (Standard Deviation of age-adjusted residuals within cohort)

**Supplementary Table 4A: Mean estimated EDSS change stratified by GFAP subgroup**

| GFAP-subgroup | Period | Estimated Mean Change | SE | p-value* | 95% CI: Lower bound | 95% CI upper bound | Effect size |
| --- | --- | --- | --- | --- | --- | --- | --- |
| ≥78.7 pg/ml | V1-V0 | 0.06 | 0.06 | 0.706 | -0.10 | 0.23 | 0.02 |
|  | V2-V0 | 0.13 | 0.11 | 0.691 | -0.19 | 0.45 | 0.08 |
|  | V3-V0 | 0.19 | 0.17 | 0.634 | -0.29 | 0.68 | 0.10 |
|  | V4-V0 | 0.26 | 0.23 | 0.561 | -0.39 | 0.90 | 0.12 |
|  | V5-V0 | 0.32 | 0.29 | 0.441 | -0.48 | 1.13 | 0.13 |
|  | V6-V0 | 0.39 | 0.34 | 0.368 | -0.58 | 1.35 | 0.14 |
|  | V7-V0 | 0.45 | 0.40 | 0.261 | -0.68 | 1.58 | 0.16 |
| < 78.7 pg/ml | V1-V0 | 0.00 | 0.05 | 1.000 | -0.13 | 0.14 | 0.01 |
|  | V2-V0 | 0.01 | 0.10 | 1.000 | -0.27 | 0.28 | 0.01 |
|  | V3-V0 | 0.01 | 0.15 | 1.000 | -0.40 | 0.43 | 0.01 |
|  | V4-V0 | 0.01 | 0.20 | 0.985 | -0.54 | 0.57 | 0.01 |
|  | V5-V0 | 0.02 | 0.24 | 0.957 | -0.67 | 0.71 | 0.01 |
|  | V6-V0 | 0.02 | 0.29 | 0.947 | -0.81 | 0.85 | 0.01 |
|  | V7-V0 | 0.03 | 0.34 | 0.927 | -0.94 | 0.99 | 0.01 |

Data are estimated mean changes (95% CI) from linear mixed-effects models for high (≥78.7 pg/mL) vs. low (<78.7 pg/mL) baseline NfL groups.
 *p*-values are FDR-corrected. Effect sizes (Cohen's d) are shown for the overall change.

**Abbreviations:** EDSS, Expanded Disability Status Scale; FDR, false discovery rate; NfL, neurofilament light chain; SSPROM, Severity Scoring System for Progressive Myelopathy; 6MWT, Six-Minute Walk Test.

V0 Estimated marginal means for baseline; V1 Estimated marginal means for follow-up year 1; V2 Estimated marginal means for follow-up year 2; V3 Estimated marginal means for follow-up year 3; V4 Estimated marginal means for follow-up year 4; V5 Estimated marginal means for follow-up year 5; V6 Estimated marginal means for follow-up year 6; V7 Estimated marginal means for follow-up year 7

Effect size: Partial eta squared for the repeated measures analyses of variance

**Supplementary Table 4B: Mean estimated SSPROM change stratified by GFAP subgroup**

| GFAP-subgroup | Period | Estimated Mean Change | SE | p-value* | 95% CI Lower bound | 95% CI upper bound | Effect size |
| --- | --- | --- | --- | --- | --- | --- | --- |
| ≥78.7 pg/mL | V1-V0 | -1.41 | 0.37 | 0.070 | -2.46 | -0.37 | 0.45 |
|  | V2-V0 | -2.83 | 0.74 | 0.065 | -4.93 | -0.73 | 0.49 |
|  | V3-V0 | -4.24 | 1.11 | 0.054 | -7.39 | -1.10 | 0.50 |
|  | V4-V0 | -5.66 | 1.48 | 0.023 | -9.85 | -1.47 | 0.56 |
|  | V5-V0 | -7.07 | 1.85 | 0.001 | -12.32 | -1.83 | 0.60 |
|  | V6-V0 | -8.49 | 2.22 | 0.001 | -14.78 | -2.20 | 0.65 |
|  | V7-V0 | -9.90 | 2.59 | <0.001 | -17.24 | -2.57 | 0.65 |
| < 78.7 pg/mL | V1-V0 | -0.79 | 0.33 | 0.410 | -1.73 | 0.14 | 0.07 |
|  | V2-V0 | -1.59 | 0.65 | 0.346 | -3.46 | 0.28 | 0.13 |
|  | V3-V0 | -2.38 | 0.98 | 0.281 | -5.19 | 0.42 | 0.18 |
|  | V4-V0 | -3.18 | 1.31 | 0.217 | -6.92 | 0.56 | 0.24 |
|  | V5-V0 | -3.97 | 1.63 | 0.152 | -8.65 | 0.70 | 0.30 |
|  | V6-V0 | -4.77 | 1.96 | 0.088 | -10.37 | 0.84 | 0.35 |
|  | V7-V0 | -5.56 | 2.28 | 0.023 | -12.10 | 0.98 | 0.41 |

Data are estimated mean changes (95% CI) from linear mixed-effects models for high (≥78.7 pg/mL) vs. low (<78.7 pg/mL) baseline NfL groups.
 *p*-values are FDR-corrected. Effect sizes (Cohen's d) are shown for the overall change.

**Abbreviations:** EDSS, Expanded Disability Status Scale; FDR, false discovery rate; NfL, neurofilament light chain; SSPROM, Severity Scoring System for Progressive Myelopathy; 6MWT, Six-Minute Walk Test.

V0 Estimated marginal means for baseline; V1 Estimated marginal means for follow-up year 1; V2 Estimated marginal means for follow-up year 2; V3 Estimated marginal means for follow-up year 3; V4 Estimated marginal means for follow-up year 4; V5 Estimated marginal means for follow-up year 5; V6 Estimated marginal means for follow-up year 6; V7 Estimated marginal means for follow-up year 7

Effect size: Partial eta squared for the repeated measures analyses of variance

**Supplementary Table 4C: Mean estimated change 6-MWT stratified by GFAP subgroup**

| GFAP-subgroup | Period | Estimated Mean Change | SE | p-value* | 95% CI lower bound | 95% CI upper bound | Effect size |
| --- | --- | --- | --- | --- | --- | --- | --- |
| ≥78.7 pg/ml | V1-V0 | -4.45 | 4.41 | 0.410 | -16.79 | 7.90 | 0.04 |
|  | V2-V0 | -8.90 | 8.82 | 0.399 | -33.59 | 15.80 | 0.06 |
|  | V3-V0 | -13.34 | 13.23 | 0.373 | -50.38 | 23.70 | 0.08 |
|  | V4-V0 | -17.79 | 17.64 | 0.334 | -67.18 | 31.60 | 0.10 |
|  | V5-V0 | -22.24 | 22.05 | 0.282 | -83.97 | 39.50 | 0.11 |
|  | V6-V0 | -26.69 | 26.45 | 0.218 | -100.77 | 47.40 | 0.13 |
|  | V7-V0 | -31.13 | 30.86 | 0.144 | -117.56 | 55.30 | 0.14 |
| < 78.7 pg/ml | V1-V0 | -6.60 | 4.04 | 0.410 | -17.92 | 4.71 | 0.07 |
|  | V2-V0 | -13.21 | 8.09 | 0.398 | -35.84 | 9.42 | 0.10 |
|  | V3-V0 | -19.81 | 12.13 | 0.368 | -53.76 | 14.13 | 0.12 |
|  | V4-V0 | -26.42 | 16.18 | 0.323 | -71.68 | 18.84 | 0.15 |
|  | V5-V0 | -33.02 | 20.22 | 0.264 | -89.60 | 23.55 | 0.18 |
|  | V6-V0 | -39.63 | 24.27 | 0.192 | -107.52 | 28.26 | 0.20 |
|  | V7-V0 | -46.23 | 28.31 | 0.108 | -125.44 | 32.97 | 0.23 |

Data are estimated mean changes (95% CI) from linear mixed-effects models for high (≥78.7 pg/mL) vs. low (<78.7 pg/mL) baseline NfL groups.
 *p*-values are FDR-corrected. Effect sizes (Cohen's d) are shown for the overall change.

**Abbreviations:** EDSS, Expanded Disability Status Scale; FDR, false discovery rate; NfL, neurofilament light chain; SSPROM, Severity Scoring System for Progressive Myelopathy; 6MWT, Six-Minute Walk Test.

V0 Estimated marginal means for baseline; V1 Estimated marginal means for follow-up year 1; V2 Estimated marginal means for follow-up year 2; V3 Estimated marginal means for follow-up year 3; V4 Estimated marginal means for follow-up year 4; V5 Estimated marginal means for follow-up year 5; V6 Estimated marginal means for follow-up year 6; V7 Estimated marginal means for follow-up year 7

Effect size: Partial eta squared for the repeated measures analyses of variance

**Supplementary Figure 1: sNfL trajectory over time**

**Figure legend:**
Predicted plasma NfL (sNfL) concentrations across study visits (baseline to year 7) estimated using a linear mixed-effects model with visit treated as a continuous variable and random intercepts and slopes for participants. The solid line represents the model-estimated mean NfL trajectory, and the shaded area indicates the 95% confidence interval. No statistically significant linear change in plasma NfL over time was observed (p = 0.111).

**Supplementary Figure 2: sGFAP trajectory over time**

**Figure legend:**
Predicted plasma GFAP (sGFAP) concentrations across study visits (baseline to year 7) estimated using a linear mixed-effects model with visit treated as a continuous variable and random intercepts and slopes for participants. The solid line represents the model-estimated mean GFAP trajectory, and the shaded area indicates the 95% confidence interval. A statistically significant increase in plasma GFAP over time was observed (linear visit effect p = 0.0087).
